# Identification of the shared mechanisms and common biomarkers between Sjögren’s syndrome and atherosclerosis using integrated bioinformatics analysis

**DOI:** 10.3389/fmed.2023.1185303

**Published:** 2023-08-31

**Authors:** Xiaoyi Qi, Qianwen Huang, Shijia Wang, Liangxian Qiu, Xiongbiao Chen, Kunfu Ouyang, Yanjun Chen

**Affiliations:** ^1^Departments of Cardiology, Peking University Shenzhen Hospital, Shenzhen, China; ^2^Medical College, Shantou University, Shantou, China; ^3^Department of Cardiovascular Surgery, Peking University Shenzhen Hospital, Shenzhen, China

**Keywords:** Sjögren’s syndrome, atherosclerosis, bioinformatics, immune infiltration, GEO database

## Abstract

**Background:**

Sjögren’s syndrome (SS) is a chronic autoimmune disease characterized by exocrine and extra-glandular symptoms. The literature indicates that SS is an independent risk factor for atherosclerosis (AS); however, its pathophysiological mechanism remains undetermined. This investigation aimed to elucidate the crosstalk genes and pathways influencing the pathophysiology of SS and AS via bioinformatic analysis of microarray data.

**Methods:**

Microarray datasets of SS (GSE40611) and AS (GSE28829) were retrieved from the Gene Expression Omnibus (GEO) database. Differentially expressed genes (DEGs) were acquired using R software’s “limma” packages, and the functions of common DEGs were determined using Gene Ontology and Kyoto Encyclopedia analyses. The protein–protein interaction (PPI) was established using the STRING database. The hub genes were assessed via cytoHubba plug-in and validated by external validation datasets (GSE84844 for SS; GSE43292 for AS). Gene set enrichment analysis (GSEA) and immune infiltration of hub genes were also conducted.

**Results:**

Eight 8 hub genes were identified using the intersection of four topological algorithms in the PPI network. Four genes (CTSS, IRF8, CYBB, and PTPRC) were then verified as important cross-talk genes between AS and SS with an area under the curve (AUC) ≥0.7. Furthermore, the immune infiltration analysis revealed that lymphocytes and macrophages are essentially linked with the pathogenesis of AS and SS. Moreover, the shared genes were enriched in multiple metabolisms and autoimmune disease-related pathways, as evidenced by GSEA analyses.

**Conclusion:**

This is the first study to explore the common mechanism between SS and AS. Four key genes, including CTSS, CYBB, IRF8, and PTPRC, were associated with the pathogenesis of SS and AS. These hub genes and their correlation with immune cells could be a potential diagnostic and therapeutic target.

## Introduction

Sjögren’s syndrome (SS) is a chronic, heterogeneous autoimmune disease characterized by lymphocyte infiltration of exocrine glands (1, 2). The epidemiological studies have revealed that the incidence rate of SS is 6.92/100,000 person/year, and it ranks as the 2nd most prevalent autoimmune disease in China (3). SS is manifested with the functional disorder of exocrine glands, especially lacrimal and salivary glands, and extra-glandular system lesions such as pulmonary, articular, cutaneous, and neurological (4, 5). Since it is a complicated autoimmune disorder, the exact mechanism explaining the immune response against various epithelial tissues in SS patients remains undetermined. The literature indicates that the development and progression of SS may be influenced by genetic predisposition, Ebstein-Barr virus infection, and changes in the innate and adaptive immune response (2).

Atherosclerosis (AS), a common cause of cardiovascular disease, is an inflammatory disease characterized by lipid lesions in large and medium-sized arteries (6). According to the global burden of disease study, deaths from cardiovascular disease increased by 21.1% between 2007 and 2017 (7). Clinical studies have validated the association of AS with SS. According to a recent meta-analysis, SS patients have higher pulse wave velocity and intima-media thickness (8). Additionally, an observational study indicated that compared to the control group, patients with SS had a 2-fold increased risk of cerebro-cardiovascular disease (9). The precise mechanisms that coexist between SS and AS remain to be elucidated. Further research is required to confirm potentially shared processes such as an imbalance between endothelium damage and repair, increased sympathetic neural activity, enhanced interferon-1 (INF-1) signature, and decreased paraoxonase 1 (PON-1) phenotype (oxidative stress) (9).

Contemporary bioinformatics methods can assist in understanding the disease pathogenesis at the genetic level. This investigation utilized the public Gene Expression Omnibus (GEO) database to identify the shared hub genes relevant to the pathogenesis of AS and SS. Furthermore, comprehensive bioinformatics analysis was performed to determine hub genes, pathways, and immune infiltration features. Identifying common hub genes could provide insight into these diseases’ potential mechanisms and therapeutic targets.

## Materials and methods

### Microarray data

The gene expression data of SS and AS was acquired from the GEO database.^1^ The articles with data comprising: (1) at least fifteen samples, (2) adult human gene expression profiles, and (3) SS patients and healthy control samples were included in this investigation. Gene expression datasets of SS (GSE40611) were based on the GPL6244 platform, containing parotid gland tissues harvested from 17 primary-SS (pSS) patients, 14 non-pSS sicca patients, and 18 controls (10). As an external validation set, GSE84844 contains whole blood transcriptomes from 30 healthy and pSS individuals, respectively (11). The GPL570 platform acquired gene expression data of AS (GSE28829) contains 13 samples from early atherosclerotic plaque and 16 from advanced atherosclerotic lesions (12). The GSE49332 dataset of 32 hypertension patients with paired pieces of carotid endarterectomy was utilized to validate the diagnostic value (13).

### Identification of differentially expressed genes

Normalization and log2 transformation were performed on the raw dataset using R (4.2.2) software. The “limma” package was used for selecting the DEGs in GSE40611 and GSE28829, with the following criteria: |log2 Fold change (FC)| > 0.5 and value of *p* <0.01. The DEGs were visualized using a volcano map and heatmap.

### Functional enrichment analysis

Functional enrichment analysis was performed using the R package “clusterProfiler,” and visualized using “the ggplot2” package. The Gene Ontology (GO) system provides structured, computable information about the functions of genes and their products (14). The Kyoto Encyclopedia of Genes and Genomes (KEGG) is an extensively used database for investigating gene functions (15). *p*- and *q*-value cutoffs were set at 0.05, respectively. GO and KEGG analyses were performed based on the intersection of SS and AS DEGs.

### Protein–protein interaction network construction and identification of hub genes

Based on the identified common DEGs, PPI networks were built using the STRING database^2^ and visualized by Cytoscape 3.9.1. (16, 17). The minimum required interaction score was 0.4. The hub genes were identified based on their network characteristics via CytoHubba, a plug-in for Cytoscape software. Four algorithms in CytoHubba were selected to calculate the top 10 hub genes and determine the final hub genes by the intersection of outcomes. Additionally, using MCODE, a Cytoscape plug-in, the significant modules of core genes were identified from the PPI network complex. The criteria were as follows: degree cutoff = 2, node score cutoff = 0.2, K-core = 2, and max. depth = 100.

### Validation of candidate biomarkers expression and ROC curve

The identified hub gene expression levels were validated in GSE84884 for SS and GSE43292 for AS. The Wilcoxon test was utilized to compare the two datasets. A *p*-value <0.05 was used as the significance threshold. Receiver Operating Characteristic Curves were generated to assess hub genes’ predictive value via the pROC package in R software. Additionally, DGIdb 3.0^3^ was used to predict potential medicines target hub genes.

### Immune infiltration analysis

A computational algorithm, CIBERSORT, was adopted to evaluate immune cell proportion in AS and SS. It resolves immune cell composition by deconvolution based on gene expression profile. 22 types of immune cells were quantified using the CIBERSORT R package (18) and the LM22 gene signature from CIBERSORT’s website.^4^ The proportion of each immune cell type was visualized using a barplot. A boxplot compared the immune cell proportions in the diseased and control groups. The correlation between the hub gene and infiltrating immune cells was elucidated using the ggplot2 R packages.

### Gene set enrichment analysis

After identifying the hub genes, single-gene GSEA analysis was performed in AS and SS using the “clusterProfiler” package. Based on the median expression of the hub gene, AS or SS patients were categorized into high- and low-expression groups. GSEA^5^ was used to calculate the enrichment score of gene sets and discover different function phenotypes. GSEA was also used for comparing the biological pathways between the two groups. The c5.go.bp.v7.5.1.entrez.gmt gene set was downloaded as a reference. Enriched gene sets with nominal *p* values of <0.05, |normalized enrichment scores (NES) | > 1, and false positive rate (FDR) *q* values of <0.05 were considered significant. The top 5 activating and inhibiting pathways of each hub gene in two diseases were depicted by the Enrichplot.

### Statistical analysis

All statistical analyses were performed using R software (version 4.2.2). The student’s *t*-test assessed differences between the two groups if the variables were normally distributed. Otherwise, the Wilcoxon test would be used. A *p*-value <0.05 was considered significant.

## Results

### Identification of common differentially expressed genes

**Figure 1:**
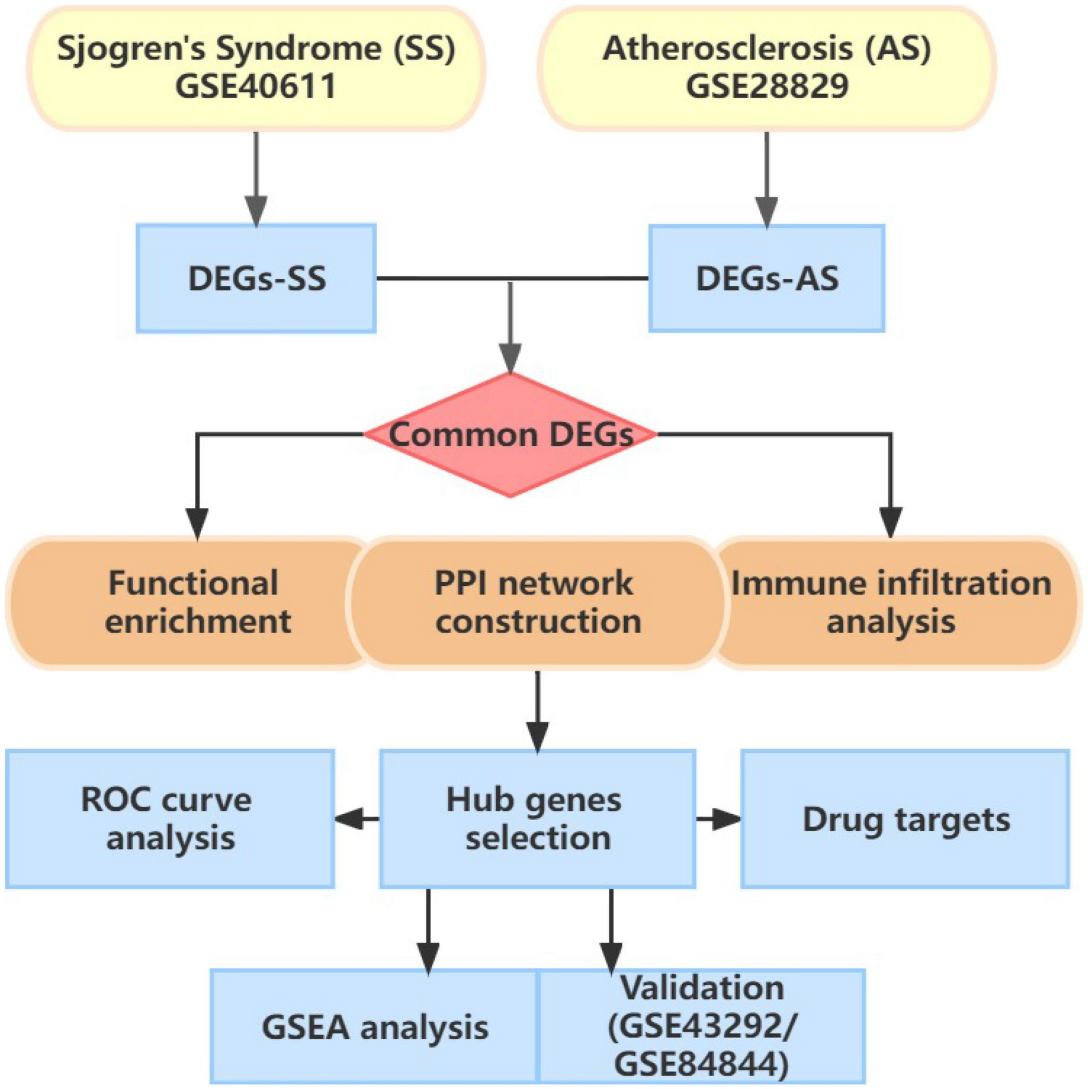
Flowchart of study design.

**Figure 2:**
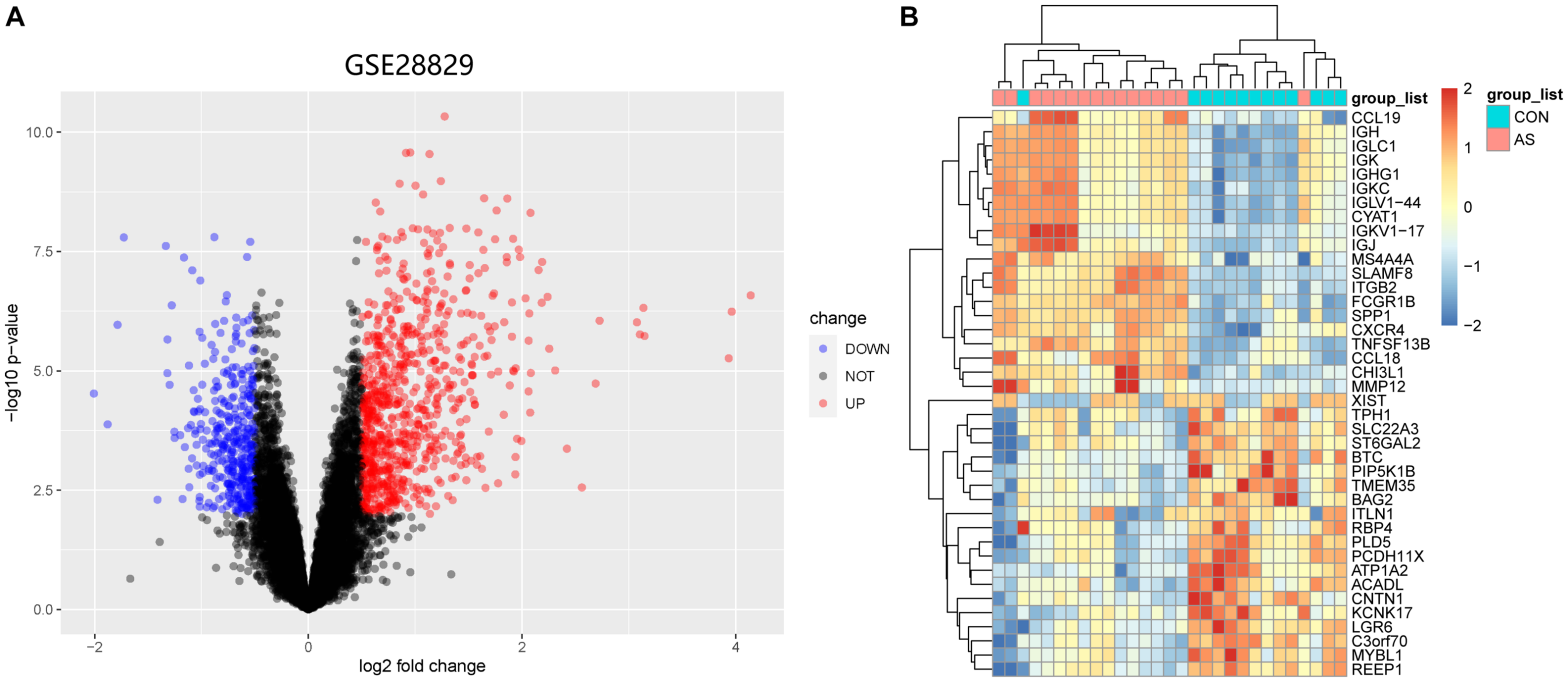
Volcano plot and Heatmap of the DEGs identified from GSE28829. **(A)** Volcano map of DEGs from GSE28829. **(B)** Heatmap of DEGs from GSE28829.

**Figure 3:**
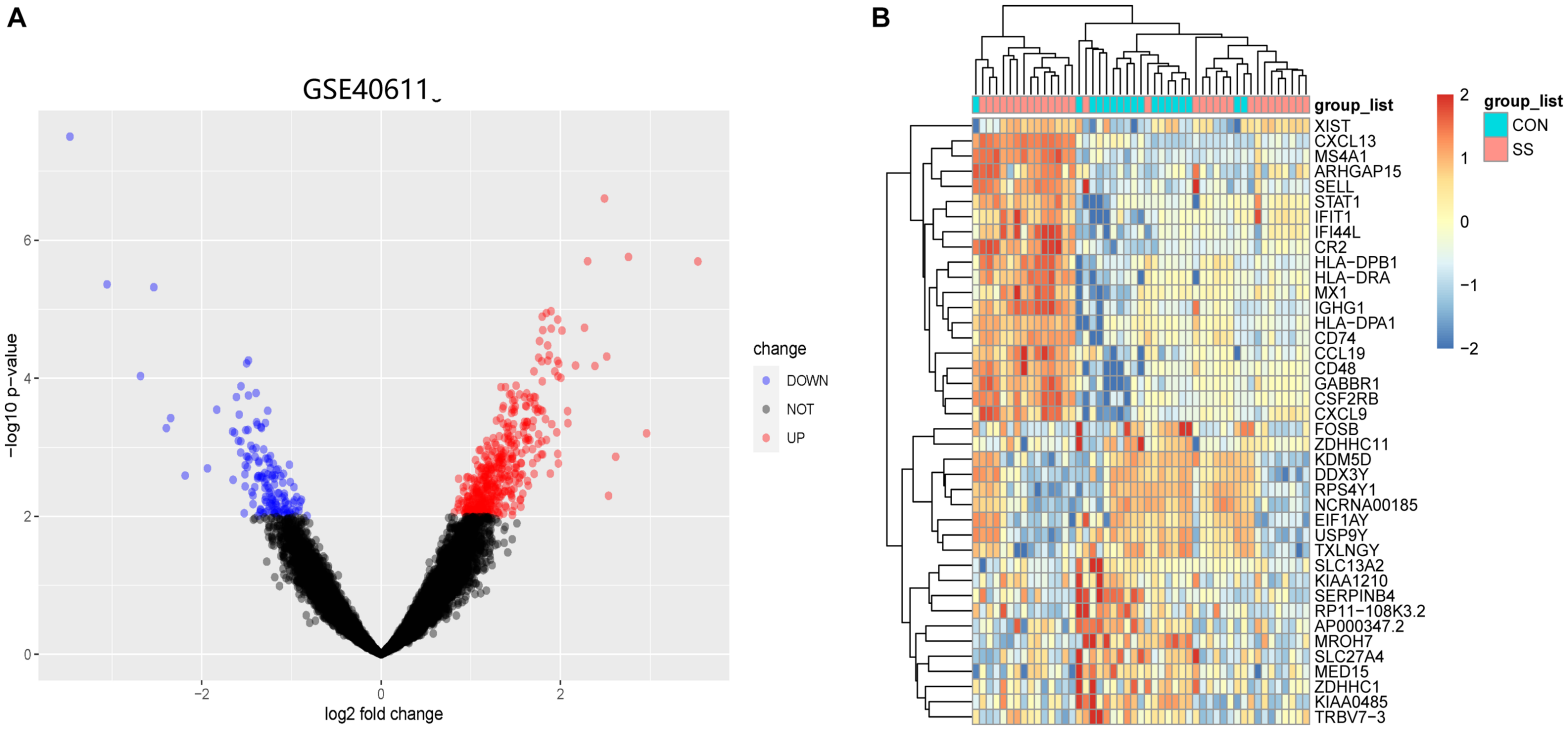
Volcano plot and Heatmap of the DEGs identified from GSE40611. **(A)** Volcano map of DEGs from GSE40611. **(B)** Heatmap of DEGs from GSE40611.

**Figure 4:**
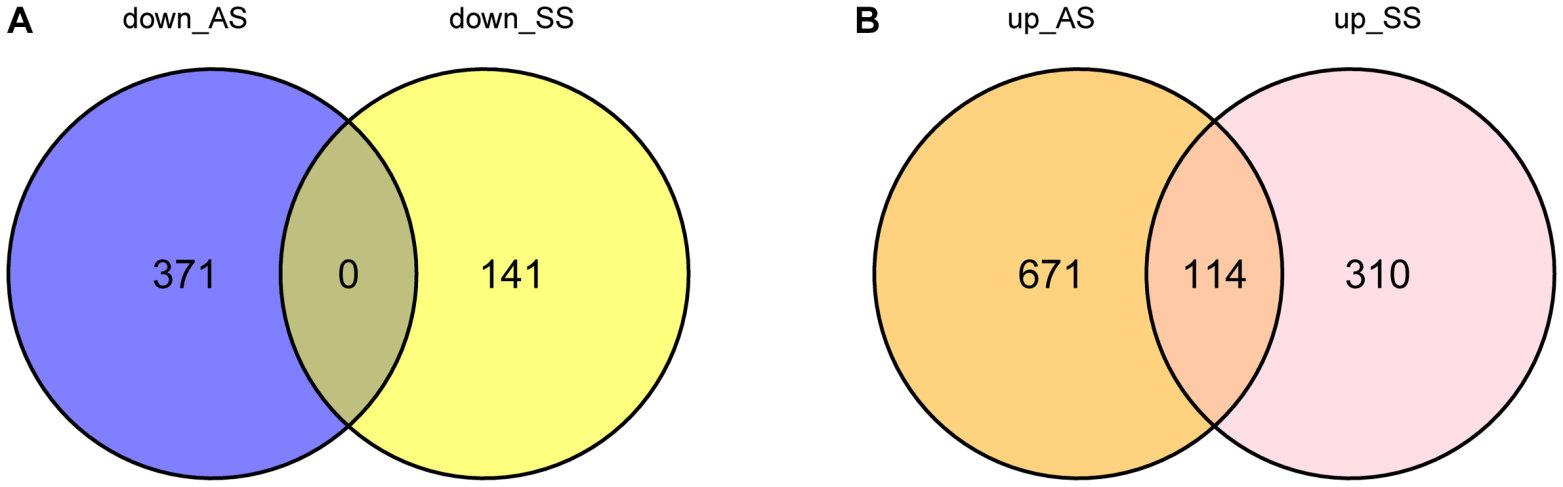
Venn plot of common DEGs between SS and AS. Where DEGs: differentially expressed genes, down_AS: downregulated DEGs in AS, down_SS: downregulated DEGs in SS, up_AS: upregulated DEGs in AS, and up_SS: upregulated DEGs in SS. **(A)** Intersection of down-regulated DEGs; **(B)** Intersection of up-regulated DEGs.

Figure 1 illustrates the flowchart of the study design. AS (GSE28892) and SS (GSE40611) microarray datasheets were downloaded from the GEO database. After screening with the criteria of -value<0.01 and |log2FC| > 0.05, 1,156 AS-DEGs (785 upregulated, 371 downregulated) and 565 SS-DEGs (424 upregulated, 141 downregulated) were identified (Figures 2, 3). By the intersecting upregulated and downregulated DEGs of SS and AS, 114 commonly upregulated DEGs were identified (Figure 4).

### Functional enrichment analysis

**Figure 5:**
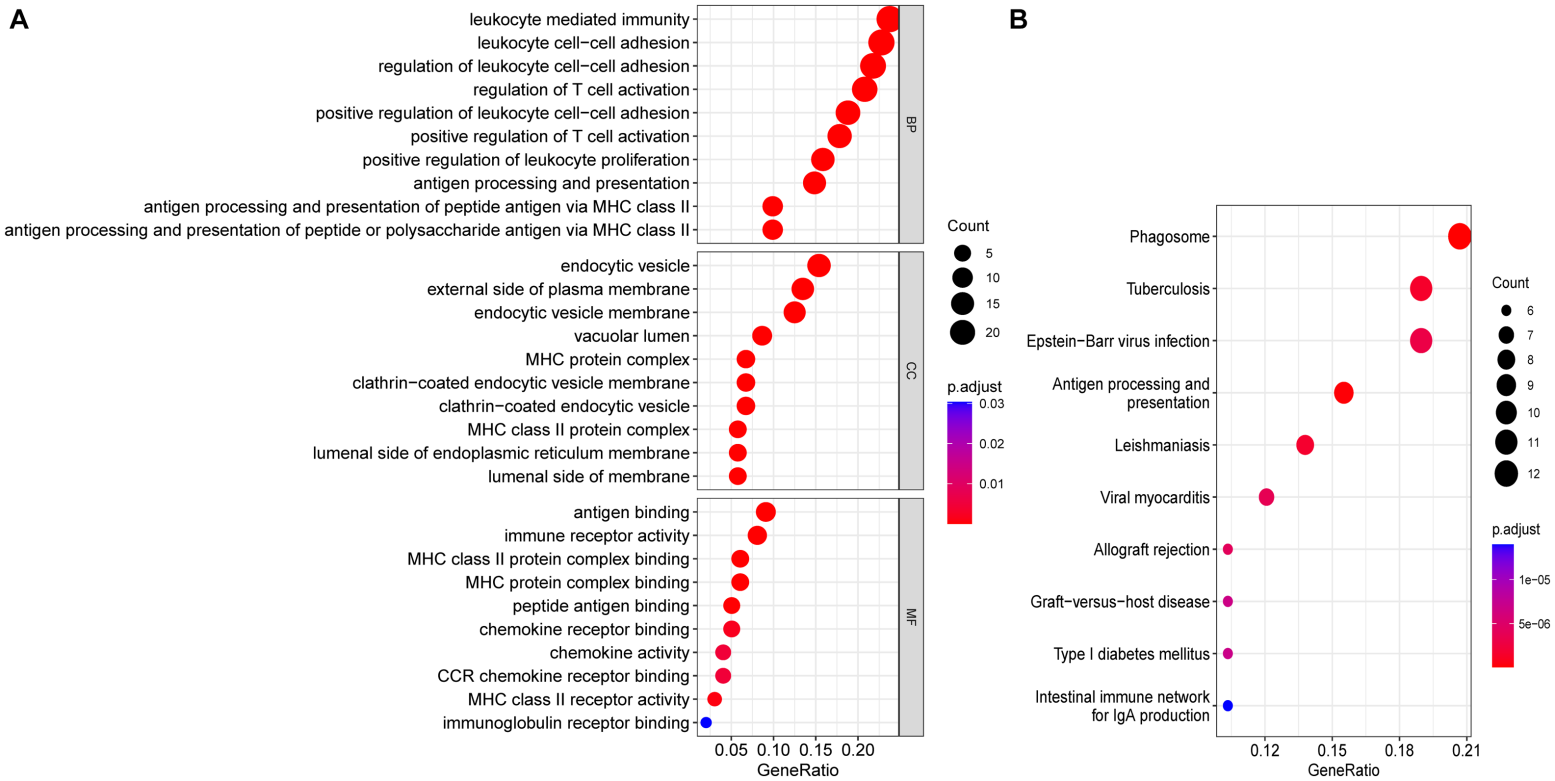
Functional enrichment analyses of common DEGs. **(A)** Bubble plot of GO enrichment analysis of common DEGs. **(B)** Bubble plot of KEGG enrichment analysis of common DEGs. Bubble size represents the number of enriched DEGs, and color represents the enrichment significance of DEGs. *p* < 0.05 and *q* < 0.05 were considered significantly enriched. DEGs, differentially expressed genes; GO, gene ontology; KEGG, kyoto encyclopedia.

Functional enrichment analyses were performed to explore the function of 114 common DEGs (Figures 5A,B). The GO enrichment pathways were associated with biological process (BP), cell component (CC), and molecular function (MF). The BP genes were mainly enriched in immune-related biological processes, such as leukocyte-mediated immunity, leukocyte cell–cell adhesion, T-cell activation regulation, and positive T-cell activation regulation. The CC genes were enriched in the endocytic vesicle, external plasma membrane region, endocytic vesicle membrane, and vacuolar lumen. Whereas the MF-related genes were enriched in immune receptor activity and antigen, chemokine receptor, and peptide bindings pathways. KEGG pathway analysis revealed that the genes were significantly enriched in pathways including phagosome, tuberculosis, Epstein–Barr virus infection, and antigen processing and presentation. These results indicated that SS and AS essentially involve immune regulation and inflammation.

### Protein–protein interaction network analysis and hub genes selection

**Figure 6:**
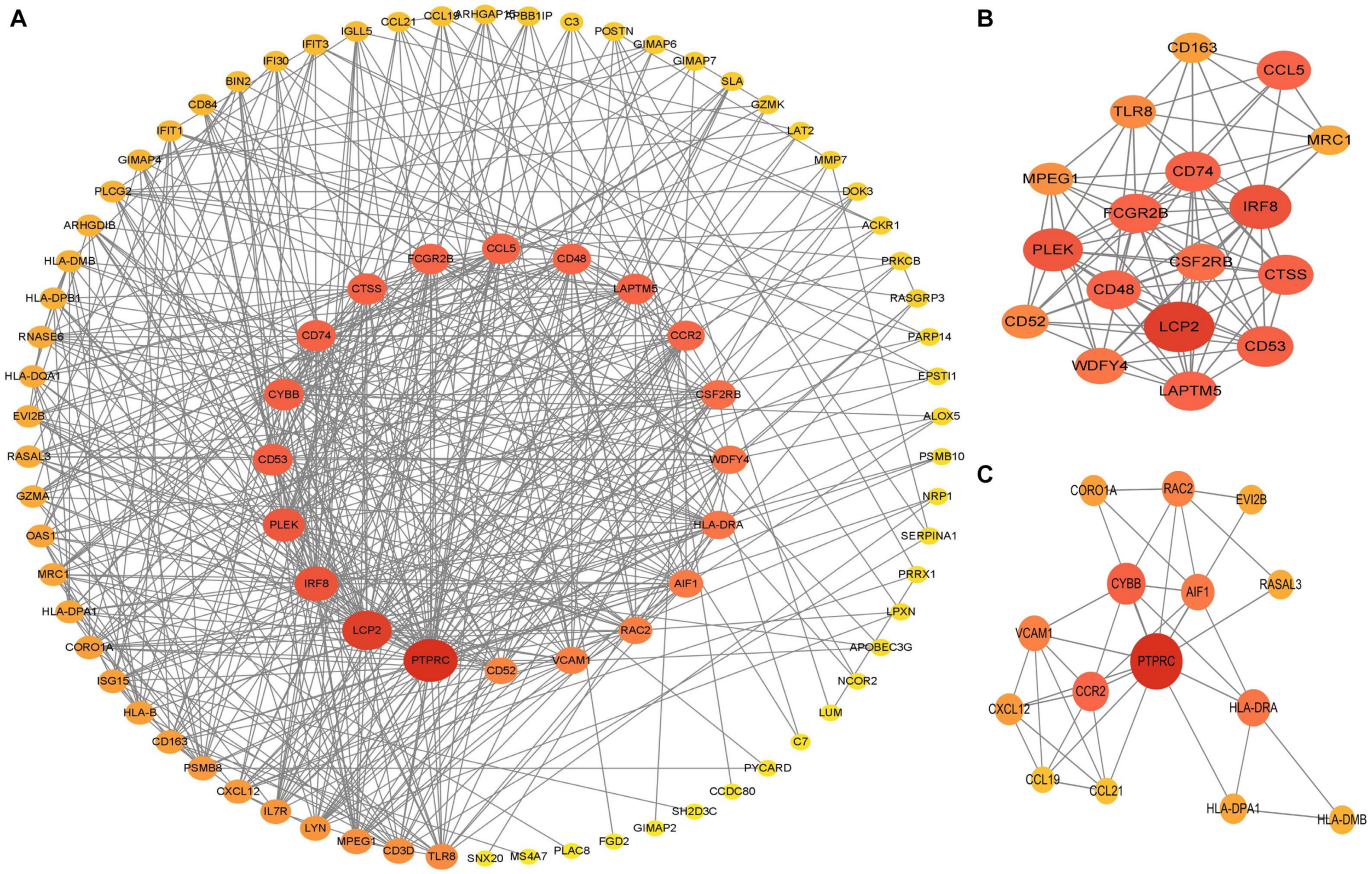
PPI networks of common DEGs. **(A)** PPI network of 66 DEGs. The color and size of nodes were arranged according to degree score. **(B,C)** Top two gene clustering modules in MCODE analysis. PPI protein–protein interaction.

**Figure 7:**
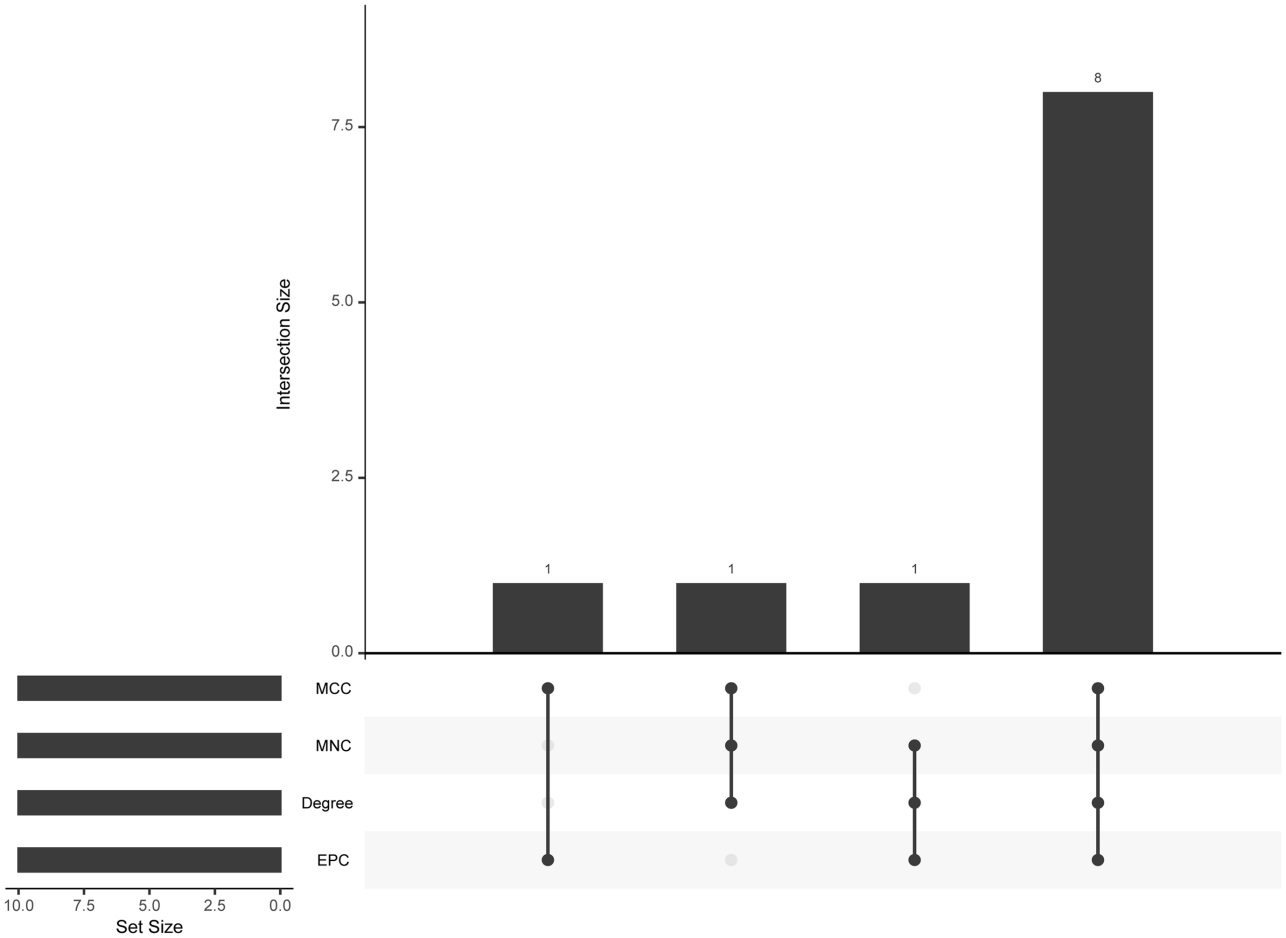
The upset plot of four algorithms characteristics in the PPI network (MCC, MNC, Degree, and EPC).

**Figure 8:**
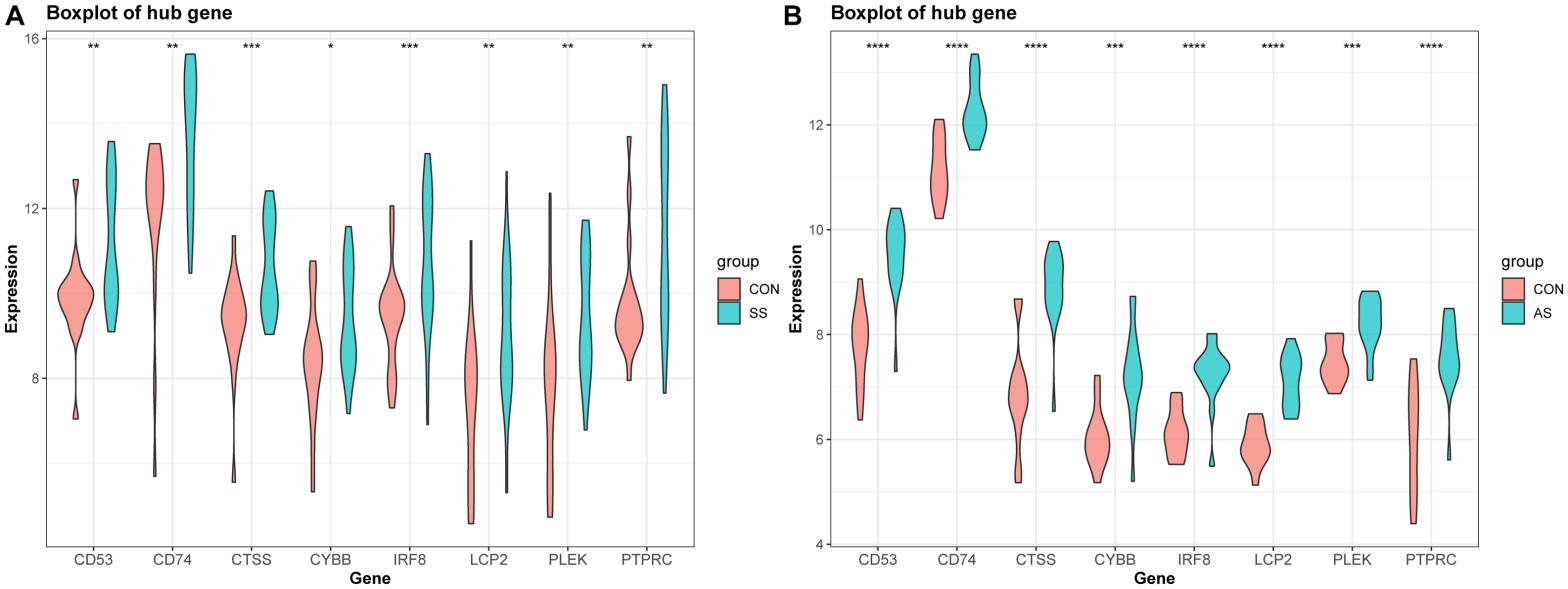
Validation of hub gene expression in AS and SS. **(A)** Boxplot of hub gene expression in SS and control group. **(B)** Boxplot of hub gene expression in AS and control group.

The PPI network (interaction score = 0.7) was established using STRING and Cytoscape software to identify the correlations between proteins encoded by SS and AS-shared DEGs, with 86 nodes and 529 edges (Figure 6A). Moreover, two functional modules were constructed using the MCODE plug-in of Cytoscape. Module 1 included 17 nodes, 92 edges, and a cluster score of 11.5. Module 2 indicated 15 nodes, 38 edges, cluster score of 5.429 (Figures 6B,C). CytoHubba, a plug-in of Cytoscape, was used to identify the hub genes in common DEGs. Based on four topological algorithms (MCC, Degree, MNC, and EPC), four groups of the top 10 highly connected genes were obtained. After assessing the overlapping genes, 8 hub genes were identified: CD53, CD74, CTSS, CYBB, IRF8, LCP2, PLEK, and PTPRC (Figure 7). The expression data indicated that in both diseases, all the hub genes were upregulated (Figure 8).

### Identification of diagnostic value of hub genes and drug targets

**Figure 9:**
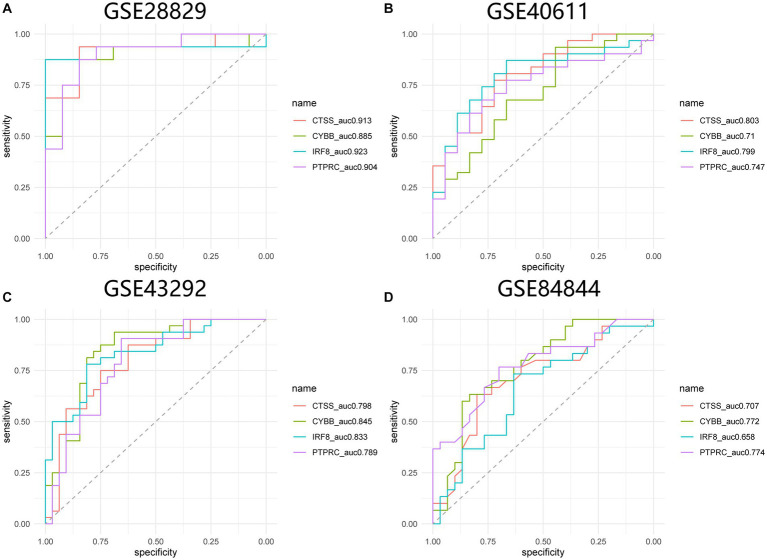
ROC curves for hub genes in SS and AS. **(A)** ROC curves for hub genes in GSE28829. **(B)** ROC curves for hub genes in GSE40611. **(C)** ROC curves for hub genes in GSE43292. **(D)** ROC curves for hub genes in GSE84844. ROC receiver operating characteristic.

**Figure 10:**
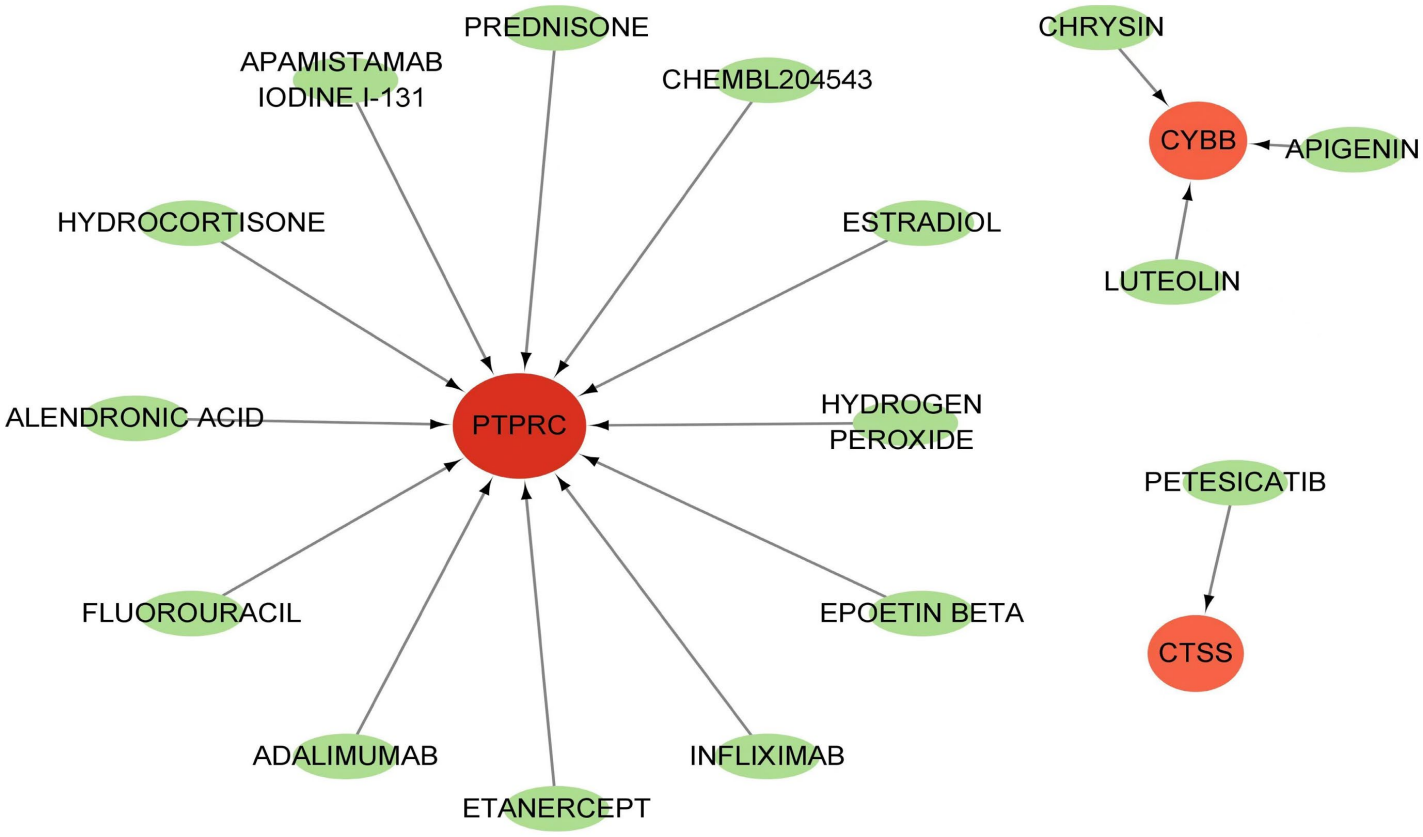
Drug target for hub genes.

GSE84884 and GSE43292 datasets were used to validate SS and AS datasets, respectively (Supplementary Figure S1). The results showed four hub genes were significantly differentially expressed in AS and SS than in the control group and were identified as final hub genes (CTSS, CYBB, IRF8, and PTPRC). pROC R package was utilized to assess the diagnostic accuracy of four hub genes (Figure 9). For AS, area under the curve (AUC) values of all the hub genes were > 70%, from highest to lowest: IRF8 (0.923), CTSS (0.913), PTPRC (0.904), and CYBB (0.885). In SS, the hub genes indicated high prediction values, from highest to lowest; CTSS (0.803), IRF8 (0.799), PTPRC (0.747), and CYBB (0.71). In the SS (GSE84884) and AS (GSE43292) validation datasets, the hub gene’s AUC value was ≥70%, confirming the result’s consistency. Furthermore, potential drugs that could mitigate the expression of hub genes were investigated from the DGIdb database (Figure 10). Searching for the DGIdb database identified three hub genes with drug target information. 10 drugs/compounds targeted PTPRC, 1 targeted CTSS, and 3 targeted CYBB. According to the results, Petesicatib was considered an inhibitor of CTSS, while the drug-gene interactions of others were unclear.

### Immune cell infiltration and association with hub genes

**Figure 11:**
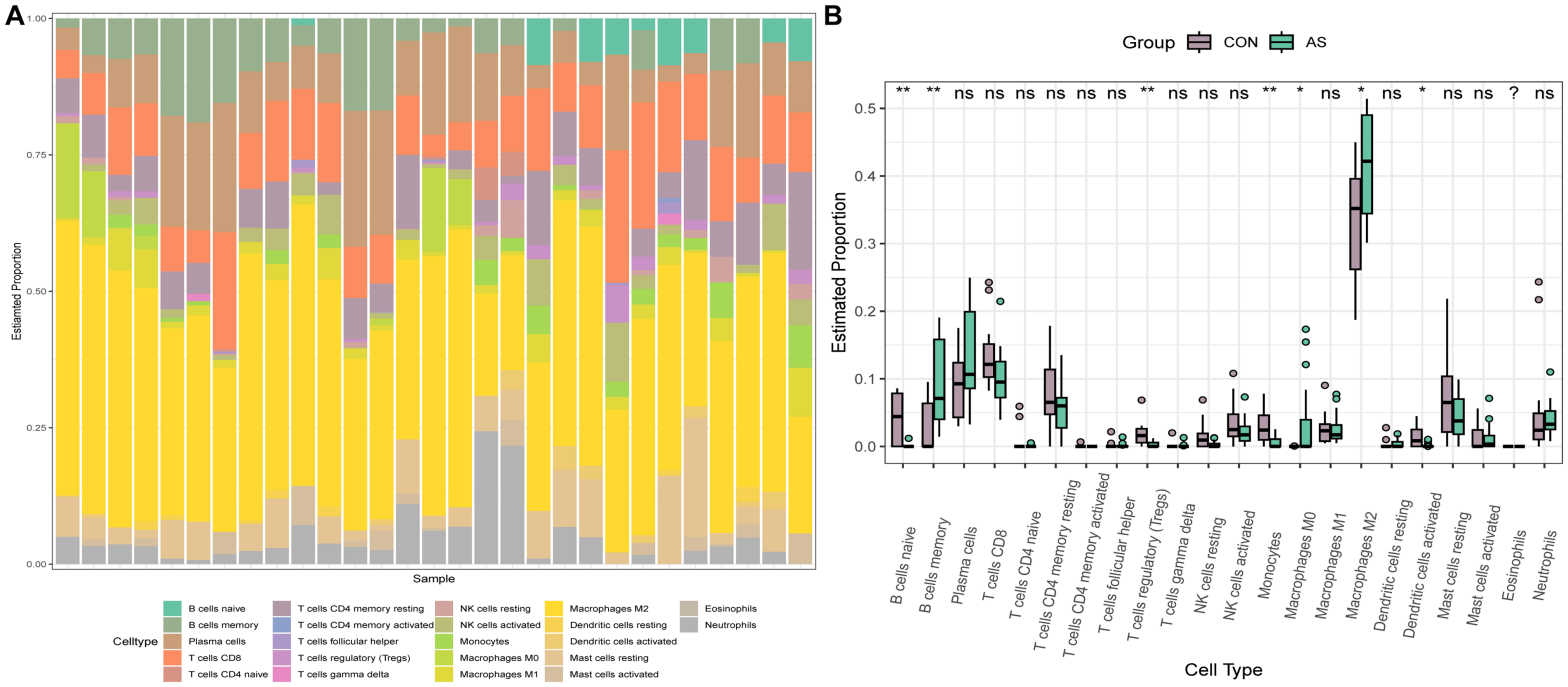
Immune infiltration analysis of AS. **(A)** Barplot of the 22 immune cells proportion. **(B)** Comparison of immune cell proportion between AS and controls (Willcoxon’s test). *p < 0.05, **p < 0.01, ***p < 0.001.

**Figure 12:**
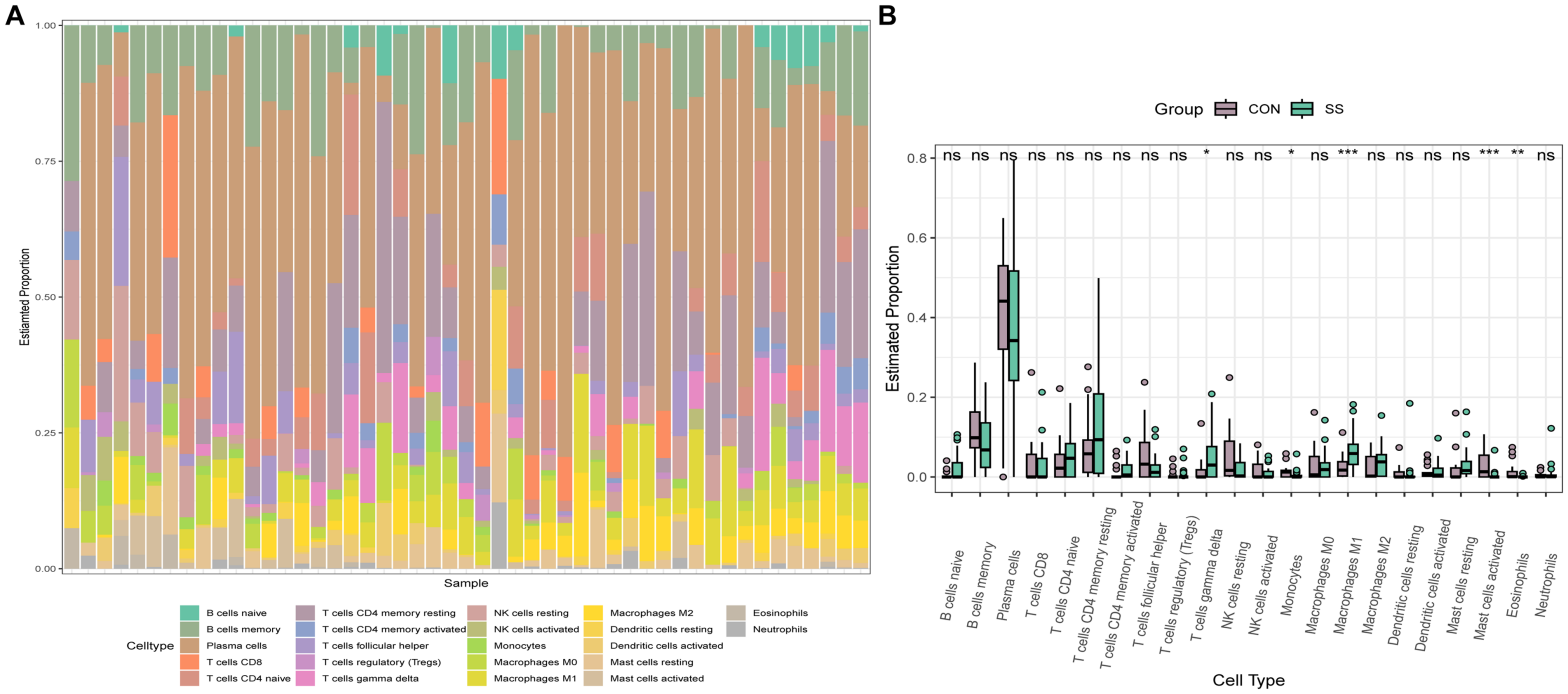
Immune infiltration analysis of SS. **(A)** Barplot of the 22 immune cells proportion. **(B)** Comparison of immune cell proportion between SS and controls (Willcoxon’s test). *p < 0.05, **p < 0.01, ***p < 0.001.

**Figure 13:**
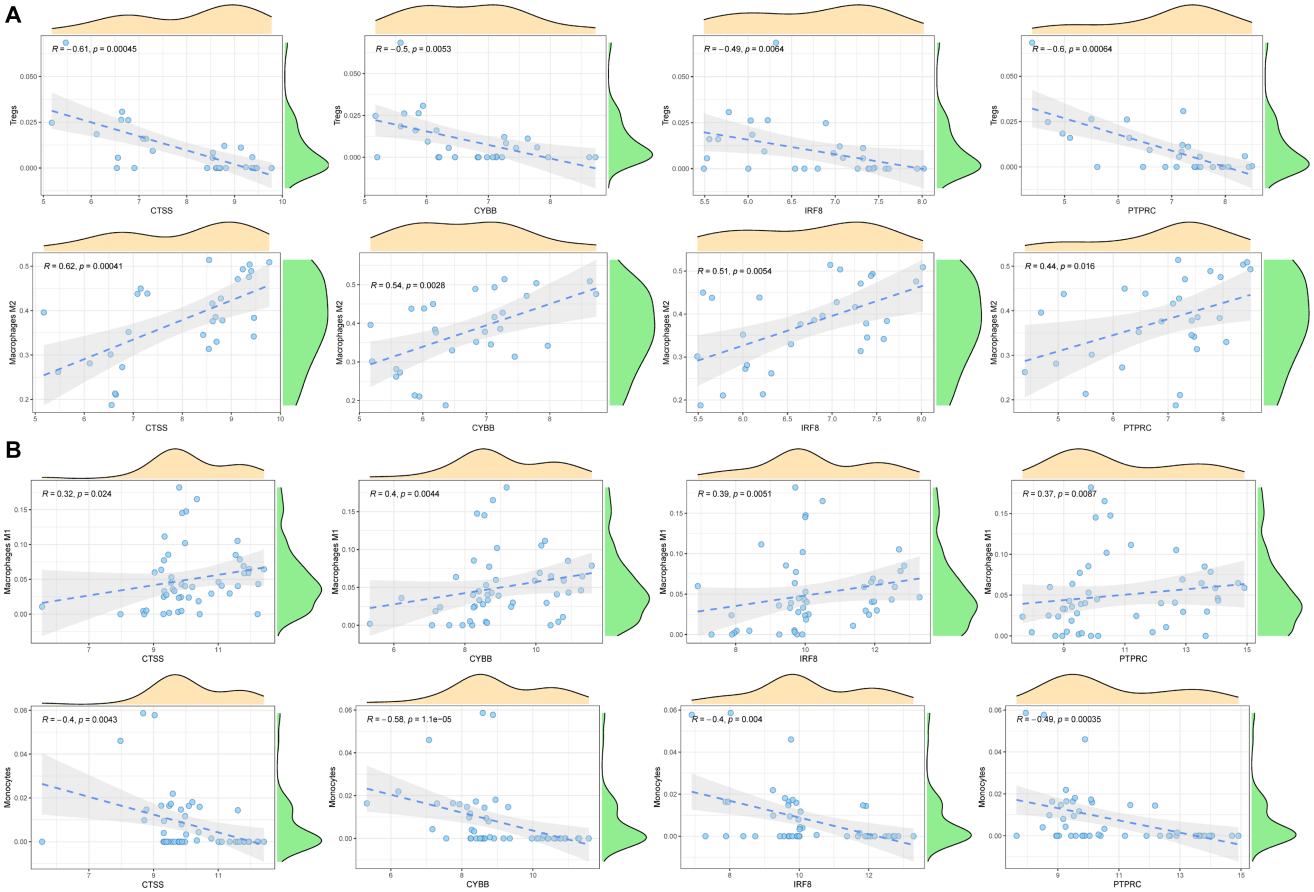
Correlation analysis between hub genes and immune cells. **(A)** Correlation analysis between hub genes and immune cells in AS. **(B)** Correlation analysis between hub genes and immune cells in SS.

To explore the immune cell infiltration profile, the CIBERSORT algorithm was applied to analyze the quantity of 22 immune cells in GSE40611 and GSE28829 (Figures 11, 12). In SS, the proportion of T cells gamma delta, and macrophage M1 was increased, whereas that of monocytes, mast cells activated, and eosinophils was decreased. AS indicated increased B cells memory and M0 and M2 macrophages than control, but decreased B cells naïve, regulatory T (T_reg_) cells, monocyte, and dendritic cells. Correlation analysis was conducted between hub genes and immune cells (Figures 13A,B). The Spearman correlation test was used to determine the association, which revealed that, in SS, all the hub genes were negatively associated with monocytes but positively associated with macrophages M1, whereas, in AS, all hub genes were positively correlated with macrophages M2, while negatively correlated with T_regs_. The above analysis revealed that macrophages and lymphocytes were involved in the progress of AS and SS.

### GSEA analysis of hub genes

**Figure 14:**
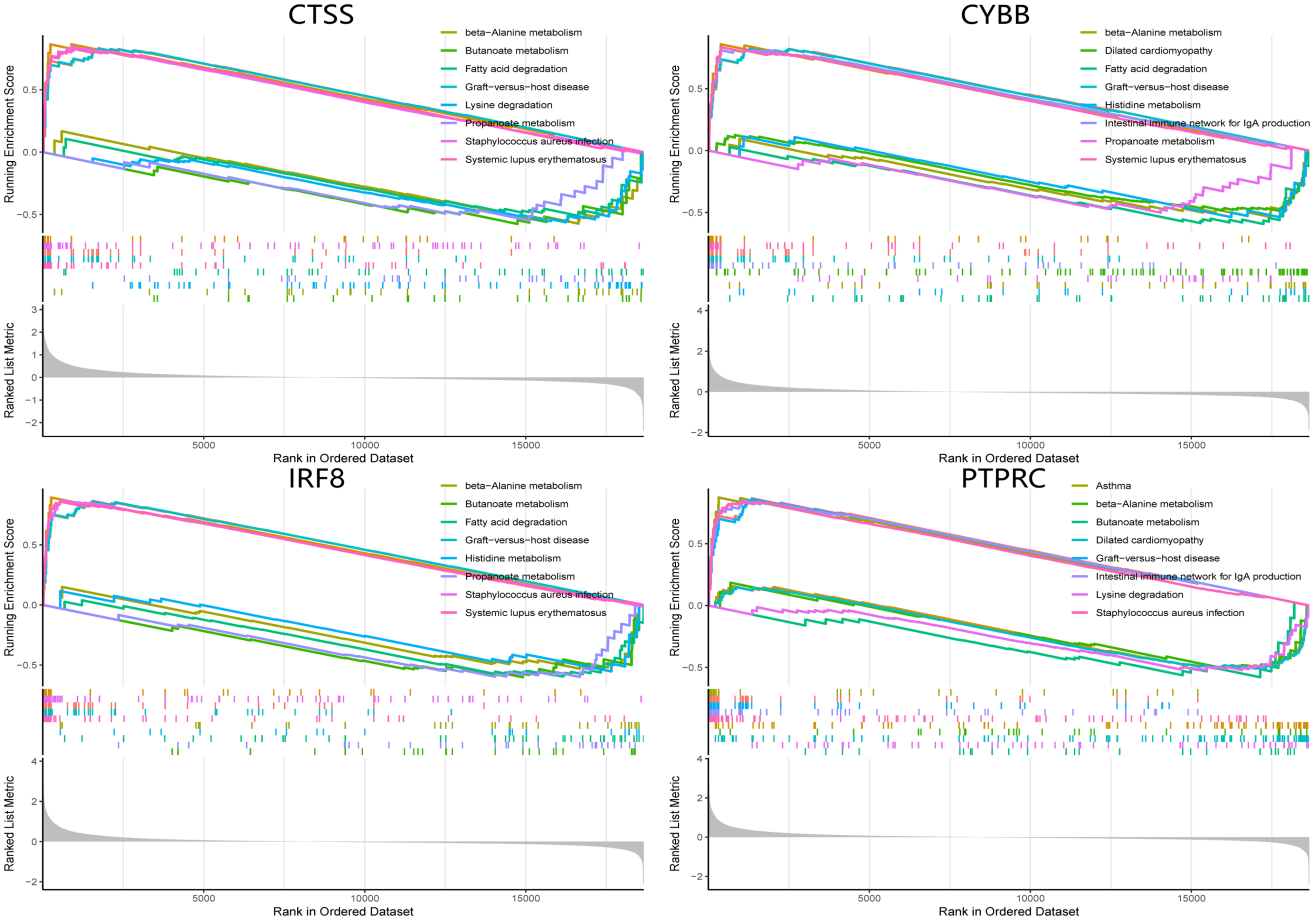
GSEA analysis of hub genes (CYBB, CTSS, IRF8, and PTPRC) in AS. GSEA Gene set enrichment analysis.

**Figure 15:**
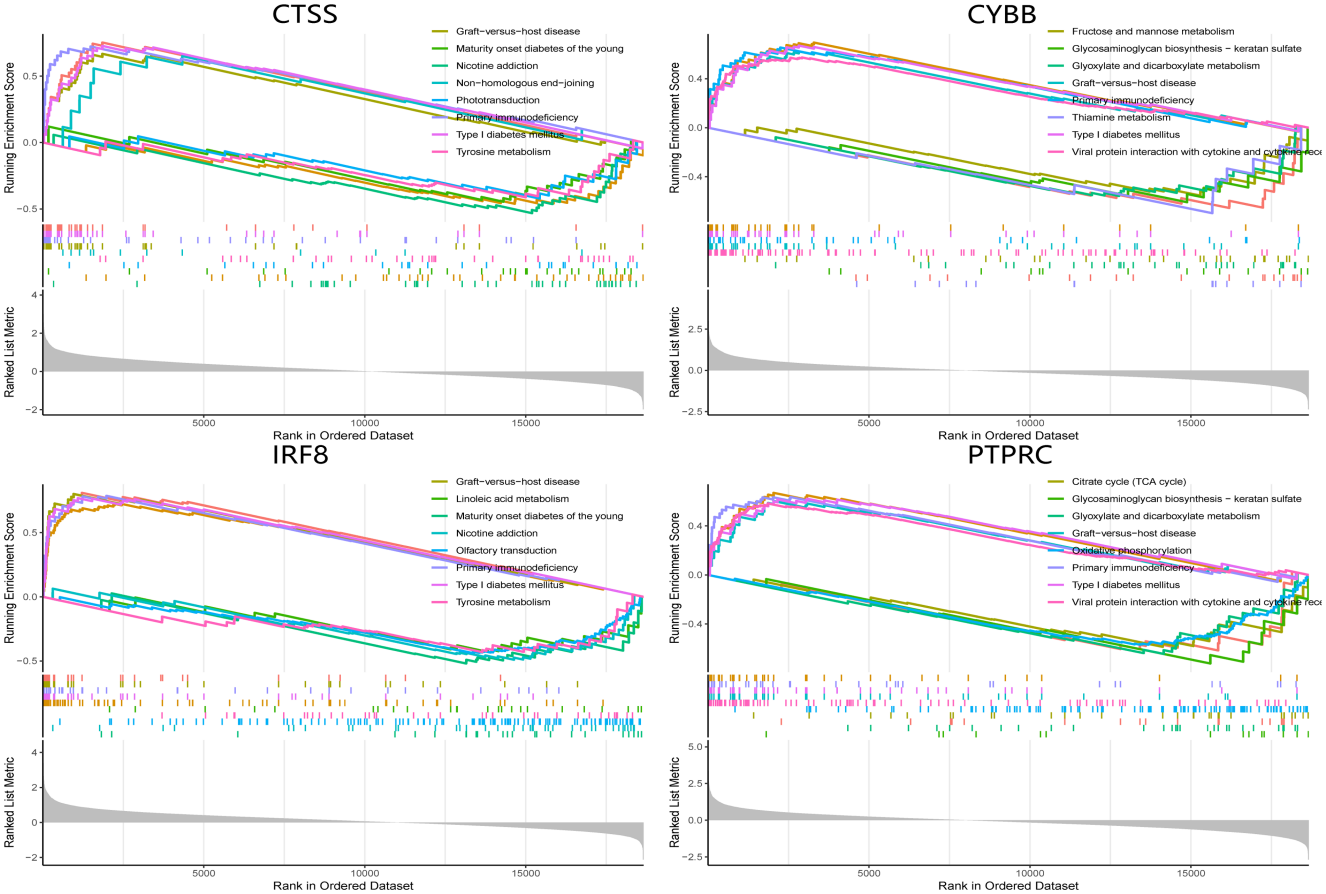
GSEA analysis of hub genes (CYBB, CTSS, IRF8, and PTPRC) in SS. GSEA Gene set enrichment analysis.

The single-gene GSEA analysis was employed on the four hub genes in SS and AS datasets (Figures 14, 15). The “GSEA” package visualized the top 5 up- and down-regulated pathways. In both disease groups, the hub genes involved metabolic pathways of beta-alanine, butanoate, fatty acid, propanoate, fructose, glyoxylate, and dicarboxylate. Furthermore, all genes were enriched in various autoimmune disease-related pathways such as maturity-onset diabetes of the young, type 1 diabetes mellitus, asthma, and systemic lupus erythematosus.

## Discussion

This investigation performed a comprehensive bioinformatics analysis to explore the common mechanism, shared pathways, and associated immune infiltration features in AS and SS for the first time. Four hub genes (CYBB, CTSS, IRF8, and PTPRC) were identified by intersecting the AS and SS DEGs and the PPI network. Further immune infiltration analysis revealed that hub genes were significantly correlated with B cells, T cells, and macrophages in the pathogenesis of both diseases. Per the GSEA enrichment analysis results, higher expression of hub genes was associated with metabolism pathways and multiple autoimmune diseases.

The clinical evidence has suggested an association between SS and AS. According to a multi-center retrospective cohort study, the pSS is frequently associated with cerebrovascular events and myocardial infarction (19). Furthermore, a population-based cohort study revealed that SS patients have an increased risk of developing early coronary artery disease (HR 1.43, 95% CI 1.09–1.86) (20). pSS is also considered an independent risk factor for subclinical AS (OR2.8, 95 percent CI1.04–7.54) even after adjusting for conventional risk variables (21). However, the investigations of the biological mechanisms between AS and SS are limited. People with SS have a higher incidence of classic cardiovascular risk factors like dyslipidemia, obesity, hypertension, and insulin resistance (22). Endothelial cell damage caused by SS was considered the precursor to AS. Gerli et al. (23) indicated that SS patients had significantly less nitrate-mediated vasodilation, and those in the subgroup with leukopenia and anti-SSB antibodies had higher levels of VCAM-1 and ICAM-1. Moreover, the thickening of intima-media in pSS patients was linked to circulating anti-SSA antibodies and leukopenia prevalence (24). Another endothelial dysfunction biomarker in SS patients is asymmetric dimethylarginine (ADMA). According to a study by Atzeni et al. (25) those with pSS had greater levels of ADMA, the main endogenous inhibitor of NO synthase that results in endothelial dysfunction. Therefore, anti-SSB antibodies and ADMA produced in SS cause endothelial cell damage. In addition to endothelial cell damage, the accompanied inflammation promotes AS progression. A cohort study confirms the presence of elevated levels of systemic inflammation markers, such as CRP, in SS patients. Additionally, a transverse study revealed that patients with pSS had a higher prevalence of carotid AS, associated with a higher level of serum calprotectin (26). Calprotectin is a protein complex with S100A8 and S100A9, which is involved in the pathophysiology of multiple inflammatory processes. Additionally, Nicaise et al. revealed that pSS patients had considerably higher serum levels of S100A8/A9, which triggered the production of IL-1B, IL-6, TNF-a, IFN-Y, IL-10, and IL-22 (27, 28). This may account for vascular inflammation in pSS patients.

Moreover, in SS patients, the immune system contributes to AS development. In the development of AS, macrophages take up OxLDL and transform into foam cells, which produce growth factors and cytokines to promote plaque formation (29). Macrophages are classified as M1 and M2. M1 macrophages could initiate and sustain inflammation, while M2 resolves inflammation (30). However, in this study, the hub genes were positively associated with M2 macrophages, contradicting previous knowledge. A recent study suggested that the M1/M2 classification system oversimplifies macrophage heterogeneity and diverse function (30). Therefore, further studies are required to investigate the role of M2 in the cardiovascular comorbidity of SS. The literature also indicated the role of macrophages in stimulating inflammatory cytokines or antigenic peptide-presenting cells to promote pSS (31). Furthermore, SS patients exhibit both macrophage subtypes. M1 macrophages exist in the early phage of SS and play a proinflammatory role, which activates CD4 + T cell differentiation and promotes submandibular gland inflammation (32). Consistently, this investigation indicated that SS patients have a higher proportion of M1 macrophages, and expression of the hub gene is positively associated with M1 macrophages. The crosstalk between the hub gene and M1 macrophages could be a potential therapeutic target for SS, requiring more supporting evidence. The result from a cohort study indicated that individuals with autoimmune diseases had an imbalanced Th17/T_reg_ ratio, with increased Th17 and decreased T_reg_ cells (33). For AS, T_reg_ cells suppress exacerbated inflammatory responses to enhance immune intolerance and homeostasis. T_regs_ maintain immune homeostasis by releasing immunosuppressive cytokines, including IL-10 and TGF-b, or directly inhibiting proinflammatory cytokine production from monocytes and macrophages. Thus, T_reg_ cells play a protective role in AS, and their deficiency is associated with a significant increase in atherosclerotic lesions (34). Consistent with the previous conclusion, the immune infiltration analysis revealed a significantly decreased proportion of T_reg_ cells in AS patients. Furthermore, the expression of hub genes was negatively correlated with the T_reg_ cell proportion. The association between the hub gene and T_reg_ cells in AS could be a potential therapeutic target for preventing cardiac events in SS patients, but it still requires further study.

This study identified four important cross-talk genes between SS and AS. PTPRC is a member of the protein tyrosine phosphatase (PTP) family, signaling molecules mediating several physiological processes, including cell growth, differentiation, and oncogenic transformation. PTPTRC is an essential T- and B-cell antigen receptor signaling pathway regulator. Moreover, it can suppress JAK kinase, which regulates cytokine receptor signaling. Huang et al. (35) identified PTPRC as a hub gene in the shared mechanism between AS and depression. Additionally, earlier bioinformatic analyses have shown that PTPRC is a critical gene in the pathophysiology of AS with psoriasis and rheumatoid arthritis (36, 37). Therefore, PTPRC is considered a typical biomarker for the concomitantly occurring autoimmune disorders and AS. T_reg_ cells are considered protective against AS development. PTPRC was indicated as DEGs of T_reg_ in a bioinformatics study (38). This study also showed that PTPRC was adversely linked with T_reg_ cells through immune infiltration analysis. PTPRC was also indicated as a DEG in the salivary glands of 52 SS patients, confirmed by linear discriminant analysis (39). In the whole-exome sequencing of 31 families with autoimmune disease history, 39 variants in immune-related genes across RA, SLE, and SS patients were concentrated on T cell receptor signaling pathways, and PTPRC is a component of this pathway (40). Results from a genome-wide DNA methylation study of naïve CD4 T cells from 11 pSS patients showed that PTPRC was hypomethylated, which can alter its transcript via an epigenetic mechanism (41). In the autoimmune-prone BXSB/MpJ-Yaa mouse model, PTPRC was confirmed to be an inflammatory cell marker (42). Therefore, PTPRC might promote SS via a mechanism associated with T cells. The immune infiltration analysis of this study indicated that PTPRC was significantly positively associated with naïve, memory-activated, and gamma delta CD4 T cells.

Cathepsin S (CTSS) is a lysosomal cysteine proteinase that degrades antigens to peptides for presenting on MHC class II molecules. Due to its elastase activity, CTSS is crucial in various lung disorders and is implicated in the pathogenesis of numerous inflammatory and autoimmune diseases. Plasma mRNA analysis of atheroma patients revealed that CTSS mRNA independently correlated with CD40 mRNA levels and may regulate atherogenesis via a CD40-mediated pathway (43). 12 hypertension patients’ vSMCs showed that IL-1β, a pro-atherogenic cytokine, induces the CTSS expression, indicating the potential role of CTSS in AS pathogenesis (44). Furthermore, a case–control study on the association between CTSS polymorphism and the risk of acute atherosclerotic infarction showed that the proportion of carriers with mutant allele lotus of CTSS was significantly higher in the disease group (45). Via bioinformatics analysis, Xu et al. (46) confirmed CTSS as a hub gene in developing plaque rupture in AS. CTSS is also indicated as a biomarker for SS patients. SS patients’ tear protein profiles showed that the median tear CTSS activity was 41.1 folds higher than healthy control subjects (47). In a NOD SS mouse model, expression of CTSS and proinflammatory cytokines (TNF, IL6, and IL1β) were significantly elevated (48). Additionally, research indicated that degradation of PRG4 by CTSS might be linked with diminished ocular surface lubrication in SS, explaining the dry eye symptom (49). Furthermore, CTSS inhibitor treatment for 2 weeks alleviated the reduction of basal tear secretion in the SS mouse model (50). Here, petesicatib was selected as a potential drug targeting CTSS. Previous clinical trials on the therapeutic effect of petesicatib in SS patients indicated negative results. Therefore, more research was required to determine whether CTSS could be a therapeutic target for SS patients.

IRF8, an IFN consensus sequence-binding protein, is a transcription factor of the IFN regulatory factor (IRF) family. The IRF family proteins bind to the IFN, stimulate response elements, and regulate the expression of IFN-α and IFN-β. IRF8 gene variations have been linked to subclinical AS in patients with autoimmune diseases (51). A cross-sectional study on patients with SLE identified IRF8 allele was associated with a higher risk of carotid plaques and increased intima-media thickness (*p* < 0.05) (52). However, clinical evidence associating cardiovascular morbidities with IRF8 in SS patients is lacking. Furthermore, studies reported that IRF8 expression markedly increased in response to mechanical injury in the carotid artery. IRF8 could also induce M1 polarization of macrophages, increasing pro-inflammatory cytokine release and promoting CVD progression (53). In VSMCs, the study demonstrated that IFNy could elevate the expression of IRF8 through a STAT1-dependent pathway, which further upregulated the production of CCL5 and resulted in impaired aortic contractility and T-cell migration (54). A review discussed the role of IRF8 as a STAT1-target gene and indicated that STAT1 triggered IRF8 upregulation, which linked with T cell receptor activity and facilitated the induction of interleukins as pro-atherogenic responses (55). For SS correlation, research showed antigen Ro52 interacts with IRF8 in IFN-y/T-cell receptor-stimulated macrophages, causing ubiquitination of IRF8, thereby enhancing its capacity to stimulate IL-12p40 expression (56). Furthermore, the role of IRF8 in regulating the immune response has also been studied. In patients with SS, the expression of IRF8 is decreased, which inhibits IRF1-mediated activation of Triam21 and affects the regulation of innate immune response (57). Additionally, IRF8 negatively modulates the expression of BAFF, a cytokine critical for developing and selecting B cells (58). As a transcription factor, IRF8 inhibits the differentiation of Tfh cells, the dysregulation of which is involved in multiple autoimmune diseases (59). Therefore, based on prior research, IRF8 could be identified as a biomarker for patients with AS and SS.

NADPH oxidase (NOX2), or CYBB, is a critical component of the membrane-bound oxidase of phagocytes that generates superoxide. The terminal component of a respiratory chain transfers single electrons from cytoplasmic NADPH across the plasma membrane to molecular oxygen on the exterior. The literature suggests that NOX2 activation in endothelial cells increased monocyte/macrophage recruitment, initiating plaque development (60). Douglas et al. (61) found that endothelial-targeted overexpression of NOX2 in ApoE−/− mice could increase superoxide production and macrophage recruitment by activating endothelial cells. An *in-vitro* study revealed that salicin-ß induced foam formation and monocyte adhesion via miR155/NOX2/NF-kB mediated VCAM-1 expression in VSMCs (62). Furthermore, bioinformatics analysis indicated CYBB as a key gene involved in carotid AS, which is consistent with the data of this study (63). On the contrary, studies investigating the role of CYBB in SS are scarce. Only one study reported that a missense variant of NOX2 could increase the risk of pSS (OR = 2.45 in Chinese, OR = 2.35 in European Americans) (64). Based on the hub gene function and related research, it is hypothesized that overexpression of these genes could dysregulate lymphocytes or macrophages and stimulate proatherogenic cytokines, contributing to the progression of AS. The study improved the understanding of the mechanism of atherosclerotic damage in SS, which could prevent comorbidities of cardiovascular diseases.

Previous reviews indicated the role of multiple inflammatory pathways involved in AS. The toll-like receptors (TLRs) signaling pathways could activate NF-kB and MAPK signaling pathways and enhance inflammatory cytokines expression to promote AS. Furthermore, increased NLRP3 inflammasome expression in immune cells could activate the maturation of IL-1ß and IL-18 to further accelerate atherosclerotic pathogenesis. Recent studies identified PCSK9 as an important player in AS. PCSK9 upregulation modulates cholesterol metabolism, increases TLR4 expression, and activates the NF-kB pathway to promote AS. Notch and Wnt pathways also progressed AS by regulating macrophage differentiation and lipid homeostasis, respectively. Consistent with the results of this study, IRF8 is involved in the TLR pathway to induce a proatherogenic response, and CYBB was confirmed earlier for its role in AS. Therefore, it was identified that PTPRC and CTSS could be used as new biomarkers for AS, which deserve further studies (65). For SS, type 1 and 2 INF and their inducible factors play a crucial role in the pathogenesis. As an IFN-Y inducible gene, CTSS was confirmed to correlate with SS in both animal models and clinical data. Moreover, IRF8 contributes to the early innate and later lymphocyte-mediated stage of SS (66). However, research associating PTPRC and CYBB with SS development is limited. Overall, this study investigated novel targets for AS and SS, except for commonly deregulated genes.

### Limitations

There are several limitations to this study. (1) The AS sample size was small. Although the hub genes were validated in a different dataset, the predictive value could be overestimated; therefore, a larger cohort study is required. (2) The baseline information for patients, such as age, gender, and comorbidity, were not provided in the clinical information of GSE28829 and GSE40611 datasets, which could result in bias in the DEGs identification. Thus, other bioinformatics analysis data are required to confirm the conclusion of this study. (3) The results need experimental validation as they are only based on bioinformatics analysis. However, consistent conclusions from previous studies have improved their reliability.

In the future, we would collect peripheral blood samples from SS or AS patients and healthy controls to validate the hub gene expression using the RT-qPCR analysis. Furthermore, cohort studies associating the hub gene expression with cardiovascular events in SS patients will also be conducted to confirm the prognostic value.

## Conclusion

This study identified four hub genes (CTSS, CYBB, IRF8, and PTPRC) via comprehensive bioinformatics analysis. Furthermore, immune infiltration alterations in AS and SS patients were also indicated, and using the DGIib database, 16 candidate drugs targeted hub genes were selected. This analysis provides potential diagnostic and therapeutic markers for patients with AS and SS.

## Data availability statement

The original contributions presented in the study are included in the article/Supplementary material, further inquiries can be directed to the corresponding author.

## Author contributions

XQ and YC designed the whole study. XQ and SW conducted the bioinformatics and statistical analyses. XQ and QH wrote the draft. KO revised and polished the manuscript. All authors contributed to the article and approved the submitted version.

## Funding

This work was supported by Shenzhen Science and Technology Innovation Foundation (no. JCYJ20180228162359914) and Guangdong-Shenzhen Joint Fund Youth Project (no. 2021A1515111110).

## Conflict of interest

The authors declare that the research was conducted in the absence of any commercial or financial relationships that could be construed as a potential conflict of interest.

## Publisher’s note

All claims expressed in this article are solely those of the authors and do not necessarily represent those of their affiliated organizations, or those of the publisher, the editors and the reviewers. Any product that may be evaluated in this article, or claim that may be made by its manufacturer, is not guaranteed or endorsed by the publisher.

## Footnotes

^1^ https://www.ncbi.nlm.nih.gov/geo/

^2^ https://cn.string-db.org/

^3^ https://www.dgidb.org

^4^ http://cibersort.stanford.edu/

^5^ http://software.broadinstitute.org/gsea/index.jsp
