# Supplementary material for: Identification of the shared mechanisms and common biomarkers between Sjögren’s syndrome and atherosclerosis using integrated bioinformatics analysis
Source: Front Med (Lausanne). 2023 Aug 31;10:1185303. doi: 10.3389/fmed.2023.1185303 (PMC10506082; doi:10.3389/fmed.2023.1185303)

Supplementary Material

Identification of the shared mechanisms and common biomarkers between Sjögren's syndrome and atherosclerosis using integrated bioinformatics analysis

Xiaoyi Qi^1^, Qianwen Huang^2^, Shijia Wang^2^, Liangxian Qiu^2^, Xiongbiao Chen^2^, Kunfu Ouyang^3^, Yanjun Chen^2^*

1 Medical College, Shantou University, Shantou, China.

2 Departments of Cardiology, Peking University Shenzhen Hospital, Shenzhen, China.

3 Department of Cardiovascular Surgery, Peking University Shenzhen Hospital, Shenzhen, China.

*** Correspondence:** Yanjun Chen, MD: chenyanjunhyd@163.com

# Supplementary Tables

Table S1. Summary of 4 GEO datasets involving SS and AS.

| No. of GSE | Platform | Samples | Disease | Group |
| --- | --- | --- | --- | --- |
| GSE28829 | GPL570 | 31patients and 18controls | Atherosclerosis | Discovery |
| GSE40611 | GPL570 | 16patients and 13controls | Sjögren's syndrome | Discovery |
| GSE43292 | GPL6244 | 32patients and 32controls | Atherosclerosis | Validation |
| GSE84844 | GPL570 | 30patients and 30controls | Sjögren's syndrome | Validation |

# Supplementary Figures

**Figure S1.** Validation of hub genes in GSE43292 and GSE84844.


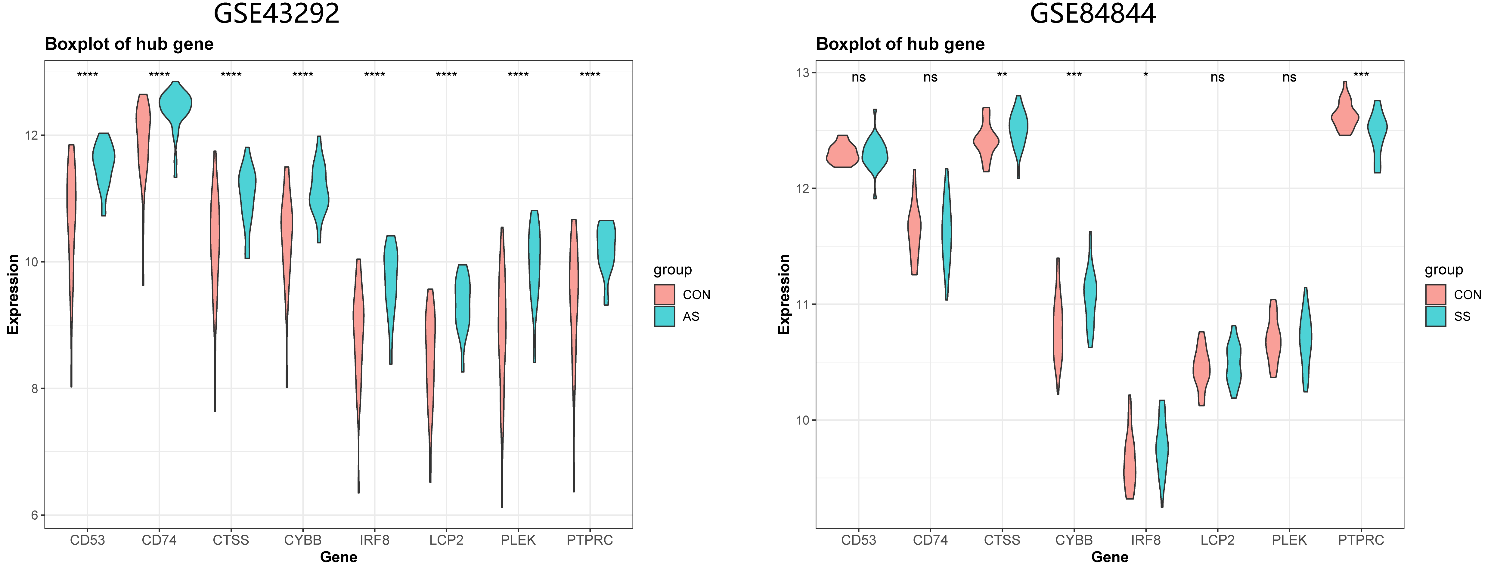

Supplement: Supplementary file 1 [file Data_Sheet_1.docx]
